# Jagged1 Expression Regulated by Notch3 and Wnt/β-catenin Signaling Pathways in Ovarian Cancer

**DOI:** 10.18632/oncotarget.127

**Published:** 2010-07-23

**Authors:** Xu Chen, Alexander Stoeck, Soo Jung Lee, Ie-Ming Shih, Michael M. Wang, Tian-Li Wang

**Affiliations:** ^1^ Departments of Gynecology and Obstetrics and Oncology, Department of Pathology, Johns Hopkins Medical Institutions, Baltimore, Maryland, USA; ^2^ Department of Neurology, University of Michigan Medical School and the VA Ann Arbor Healthcare System, Ann Arbor, Michigan, USA; ^3^ Department of Urology, Union Hospital, Tongji Medical College of Huazhong University of Science and Technology, Wuhan, China

**Keywords:** Notch3, ovarian cancer, Jagged, signaling

## Abstract

Ovarian serous carcinoma is a highly aggressive neoplastic disease in women. Our previous studies have demonstrated *Notch3* gene amplification and upregulation in many ovarian serous carcinomas and Notch pathway activity contributed to drug resistance. Among different Notch3 ligands, Jagged1 is most dominant in ovarian cancer, and Notch3 pathway activity correlated with Jagged1 expression level in ovarian carcinoma tissues. In this study, we found that Jagged1 expression depended on Notch3 pathway activation. Knockdown of either Notch3 or RBPjk, a Notchinteracting transcription factor critical in Notch signaling, suppressed Jagged1 expression in ovarian cancer cells. Moreover, Jagged1 expression was upregulated in human ovarian surface epithelial cells after ectopic expression of Notch3 intracellular domain and was upregulated in mouse epithelial cells isolated from Notch3-inducible mice after induction. We also found that inhibition of Wnt/β-catenin signaling reduced Jagged1 expression, and co-administration of shRNAs targeting both Notch3 and β-catenin reduced Jagged1 expression much more than targeting either individual gene. Taken together, our data suggested a positive regulatory loop between Notch3 and its ligand, Jagged1, in ovarian cancer cells. In addition, Wnt/β-catenin pathway activation also up-regulated Jagged1. Both mechanisms may sustain Notch3 signaling in ovarian cancer cells and contribute to the pathogenesis of ovarian carcinoma.

## INTRODUCTION

Signaling pathways that are fundamental to development and tissue differentiation are usually found to participate in the pathogenesis of human cancer [1]. For example, Hedgehog, Wnt, Notch, and TGF-β pathways were found to play critical roles in both processes. These pathways involve ligandreceptor interactions which initiate signal transduction that results in modulation of a set of downstream genes that mediate the ultimate functions of a specific pathway. However, aberrant activation of these signaling pathways may confer an advantage to tumor cells for growth in the host environment. The role of an aberrant Notch signaling pathway in human cancer is well illustrated in T-cell acute lymphocytic leukemia (T-ALL). Activating point mutations occur in more than 50% of T-ALL cases, and interstitial deletions of the extracellular portion of human Notch1 occur in another 10% due to chromosomal translocation [2, 3]. Those molecular genetic alterations result in the dysregulated expression of an oncogenic intracellular form of Notch1 [4].

By analyzing genome-wide DNA copy number changes in primary ovarian serous carcinoma, the most aggressive type of gynecologic cancer [5], we have identified a relatively frequent amplification located on *chr19p13.12*, which harbors *Notch3* [6]. We have focused on characterizing this gene because, as compared to other co-amplified genes, *Notch3* mRNA was most significantly up-regulated in amplified tumors. Notch3 (mRNA as well as protein) was found to be over-expressed in more than 50% of high-grade tumors as compared to ovarian surface epithelium. Recently, we have demonstrated that Notch3 expression is associated with recurrence postchemotherapy, probably as a result of upregulation of an ATP-dependent transporter gene, *ABCB1*, and several embryonic stem cell markers [7].

In order to determine which Notch ligand was most prevalent in ovarian cancer, we employed gene expression profiling of all known Notch ligands and found that Jagged1 was the primary Notch3 ligand in ovarian carcinoma. We observed a significant correlation between Jagged1 expression and nuclear localization of the intracellular cytoplasmic domain of Notch3 (NICD3) in ovarian serous carcinoma tissues [8]. Moreover, we found that in ovarian cancer cells, Notch3 could be co-immunoprecipitated with Jagged1 [8], and ectopic expression of NICD3 could partly rescue the growth-inhibitory effect produced by Jagged1 withdrawal [8]. These findings suggested a critical role for Jagged1-Notch3 signaling in supporting the growth and progression of ovarian cancer. While molecular genetic changes such as translocation and activating mutations provide the mechanism for constitutively activating the Notch pathway, it is not known how overexpression of Notch due to gene amplification or epigenetic upregulation sustains pathway activation. In the absence of structural alterations, increased protein levels of receptor alone may not necessarily enhance overall signaling activity unless the cognate ligand is also overexpressed. Based on our previous studies, we hypothesized that cell-cell contacts between adjacent ovarian cancer cells elicit Notch3 signaling which in turn upregulates its ligand, Jagged1, thus maintaining long-term Notch3 signaling in cancer cells.

To test this hypothesis, we used both reverse and forward approaches, based on gene knockdown and ectopic expression systems, respectively, to demonstrate that Notch3 signaling can regulate expression levels of its own ligand, Jagged1. Furthermore, because the Wnt/β-catenin pathway has been shown to affect Jagged1 expression in hair follicles [9], we also determined if Jagged1 could be regulated by the Wnt/β-catenin pathway in ovarian cancer cells.

## RESULTS

**Fig. 1:**
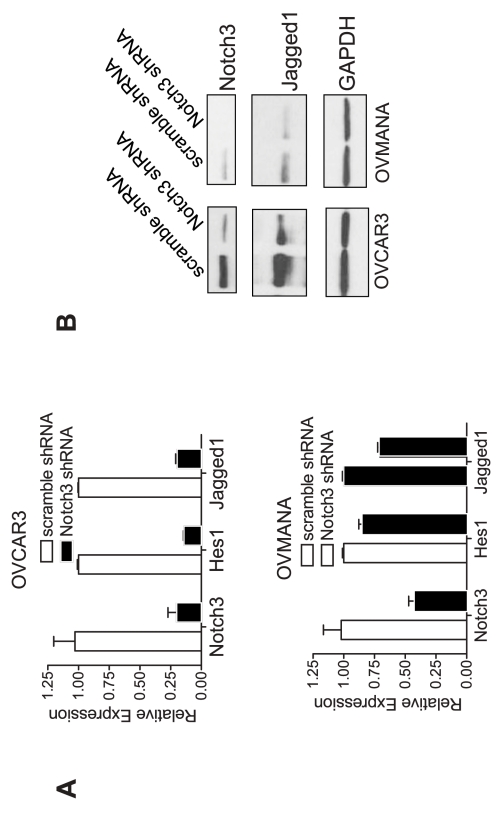
Notch3 knockdown suppresses Jagged1 expression in ovarian cancer cell lines A. Quantitatively RT-PCR performed in OVCAR3 and OVMANA cells demonstrates that Jagged1 and Hes1 transcript levels decrease after Notch3 knockdown by shRNA. B. Western blot demonstrates Jagged1 protein down-regulation after treating cells with Notch3 shRNA. Protein expression of GAPDH determined by Western blot is used as the loading control.

**Fig. 2:**
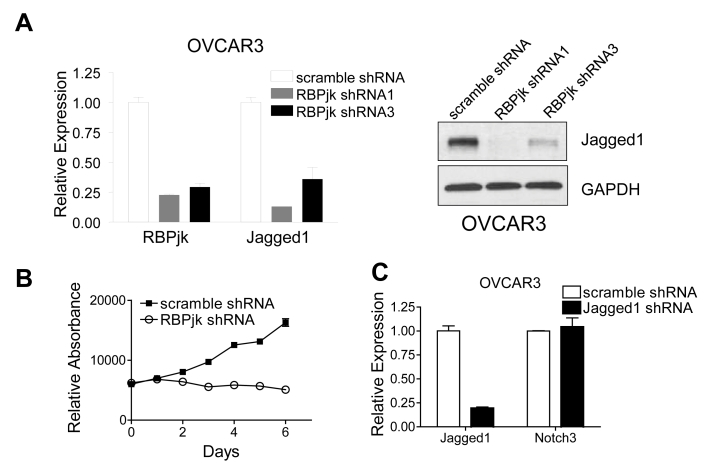
Downregulation of Jagged1 after knockdown of RBP-jκ, a mediator of the Notch3 signaling pathway, in OVCAR3 cells A. Both Jagged1 mRNA level (left panel) and protein level (right panel) decreased after *RBP-jκ* knockdown in OVCAR3 cells as detected by quantitative RT-PCR and Western blot analyses. B. Cell proliferation significantly decreases in OVCAR3 cells following *RBP-jκ* knockdown. RBP-jκ shRNA1 and shRNA was co-applied in this study. C. mRNA expression level of Notch3 is not altered by Jagged1 shRNA knockdown in OVCAR3 cells

To demonstrate whether Notch3 signaling is essential for Jagged1 expression, we compared Jagged1 mRNA and protein levels between ovarian cancer cells transfected with Notch3 specific shRNAs and control shRNA. Different ovarian cancer cell lines were tested, and OVCAR3 and OVMANA ovarian cancer cell lines were used because both cell lines expressed abundant endogenous Notch3 and Jagged1. Transfection of both OVCAR3 and OVMANA with Notch3 specific shRNAs resulted in a decrease in the transcript level of Notch3, Hes1 (an established Notch regulated gene), and Jagged1. Similarly, Western blot analysis demonstrated that Notch3 shRNAs robustly suppressed protein expression of Jagged1 as well as Notch3. The results from a representative shRNA are shown in Figure 1. To confirm that blocking expression of Notch3 is the main cause for the reduced Jagged1 expression, we used two additional approaches. First, we employed shRNAs targeting RBPjk, a gene that is critical in regulating transcription activity of the Notch signaling pathway, and second, we applied gamma secretase inhibitor (GSI), which blocks cleavage of Notch3, thus preventing generation of NICD3. RBPjk shRNA treatment decreased the expression of Jagged1 at both transcript and protein levels in ovarian cancer cells (Fig. 2A). Moreover, cell growth was significantly decreased in cells transfected with RBPjk shRNA (Fig. 2B), a result similar to that obtained by inactivating the Notch3 pathway using Notch3 gene knockdown and GSI [6]. GSI treatment also significantly reduced the transcript levels of Jagged1 and Hes1, a well-known target gene of Notch receptor (supplementary Fig. 1). These results provide evidence that Notch3 signaling activity regulates Jagged1 expression in ovarian cancer cells. In contrast, we did not observe a significant effect on the expression level of Notch3 by knocking down Jagged1, suggesting a “one-way” regulatory loop between Notch3 and Jagged1 in ovarian cancer cells (Fig. 2C).

**Fig. 3:**
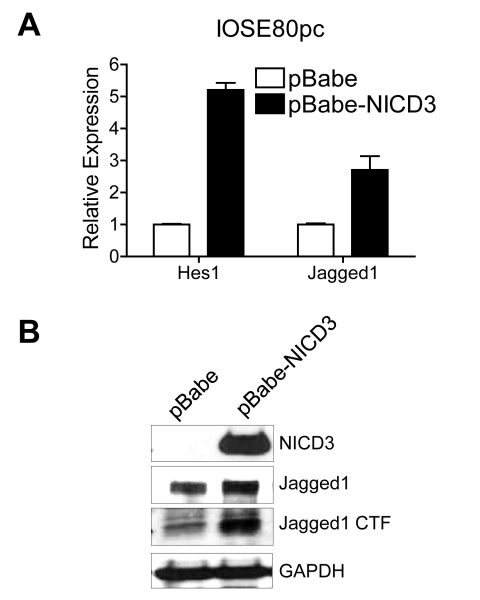
Ectopic expression of Notch3 intracellular domain upregulates Jagged-1 expression in human ovarian surface epithelial cells A. Quantitative real-time PCR shows Jagged1 mRNA upregulation after ectopic expression of Notch3 intracellular fragment (NICD3) in human ovarian surface epithelial cells, IOSE-80PC. The expression of a Notch-regulated target gene, Hes1, is used as a control. B. Western blot demonstrates that both Jagged1 full length (FL) and C-Terminal Fragment (CTF) are upregulated after ectopic expression of NICD3 in IOSE-80PC cells. GAPDH is used as the loading control.

**Fig. 4:**
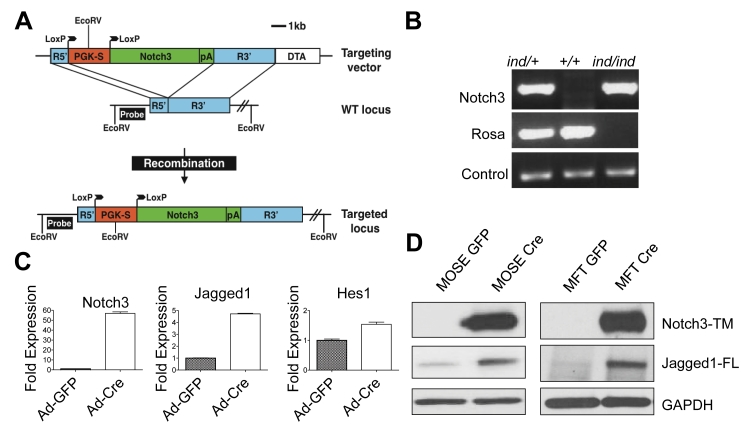
Notch3 induction increases Jagged1 expression at both mRNA and protein levels in Notch3 knockin mouse cells. A. Generation of inducible knockin mice to express human Notch3. Gene targeting strategy is illustrated. The Notch3 transgene was recombined into the ROSA26 locus of embryonic stem cells. The targeted of the locus results in a stop/polyA cassette that prevents expression in the absence of Cre recombinase. The Cre/Lox reaction (bottom) activates expression of the Notch3 cassette. B. Genotypes of Notch3 knockin mouse. The band at 340 bp corresponds to the presence of knockin

Next, using two independent approaches, we asked whether activation of the Notch3 pathway was sufficient to upregulate Jagged1 expression. First, IOSE80pc, a cell line derived from normal ovarian surface epithelium, was transfected with NICD3 retrovirus. This cell line represents the benign counterpart of ovarian carcinoma and expresses an undetectable level of NICD3. As shown in Figure 3A, IOSE-80pc cells transduced with NICD3 retrovirus expressed abundant NICD3 and expressed a significantly higher level of Jagged1 protein than the cells transduced with control virus. In addition, we also observed an increase in the expression of Jagged1 CTF, the activated form of Jagged1 in NICD3-transduced cells as compared to control virus-transduced cells. We further used Notch3 knockin mice to support the above findings. The Cre/Lox Notch3 knockin mouse was generated by inserting a Cre/Lox construct carrying full-length human Notch3 into the Rosa26 locus (Fig. 4A). Primary cultures of ovarian surface epithelial (OSE) and fallopian tube (FT) epithelial cells were established from the Notch3 knockin mouse. When Notch3 expression was induced after Ad-Cre infection (Fig 4C & D), we found that Jagged1 transcript and protein level were elevated as compared to the same cells infected with Ade-GFP control virus. The expression of a Notch3 target gene, Hes1, was also elevated in cells with induced Notch3 expression, indicating that the Notch signaling pathway was elevated.

**Fig. 5:**
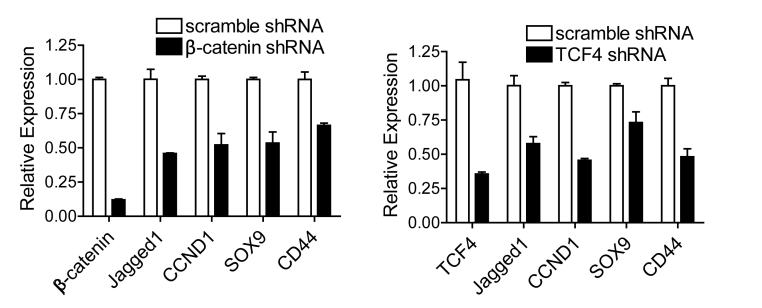
Wnt signaling pathway supports Jagged1 expression in ovarian cancer cells. Quantitative real time PCR shows a significant decrease in Jagged1 expression level after knockdown of either β-catenin (left panel) or TCF4 (right panel) in OVCAR3 cells. Well-known WNT targets including CCND1, SOX9, and CD44 are used as positive controls.

**Fig. 6:**
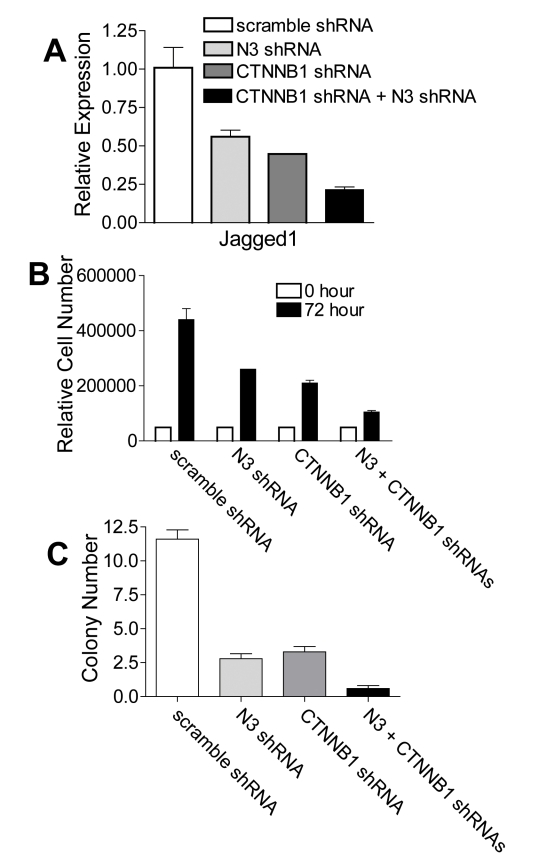
Simultaneous knockdown of both Notch3 and Wnt singling pathways in ovarian cancer cells. A. Simultaneous knockdown of both Notch3 and β-catenin in OVCAR3 cells reduces Jagged1 transcript level significantly more than knockdown of either Notch3 shRNA or β-catenin shRNA alone at the same concentration of shRNA plasmid (p<0.001). B. Cellular proliferation in OVCAR3 cells transduced by both Notch3 shRNA and β-catenin shRNA is significantly lower than in cells transduced by either shRNA virus alone (p<0.001). C. Colony formation assay performed in OVCAR3 cells demonstrates that cells transduced by both Notch3 shRNA and β-catenin shRNA have lower colony formation ability than cells transduced by either shRNA virus alone (p<0.001).

Because the Wnt/β-catenin signaling pathway has been reported to affect the expression of Jagged1 in hair follicles [9], we determined if this effect occurred in cancer cells. shRNAs targeting β-catenin and TCF-4 were used to inactivate the Wnt/β-catenin pathway in OVCAR3 cells. As shown in Figure 5, the shRNAs effectively reduced the transcript levels of β-catenin and TCF-4, respectively. More importantly, Jagged1 and several well-established Wnt regulated downstream genes including CCND1, SOX9, and CD44 were also found to be downregulated. Next, we knocked down both Notch3 and β-catenin in OVCAR3 cells and found that this reduced the Jagged1 transcript level significantly more than knockdown of either Notch1 or β-catenin alone at the same concentration of shRNA plasmid (p<0.001) (Fig. 6). Similarly, cell proliferation and colony formation potency in cells transduced by both Notch3 shRNA and β-catenin shRNA were significantly reduced compared to cells transduced by either of shRNA virus alone (p<0.001, Fig. 6 B&C).

## DISCUSSION

Maintaining optimal stoichiometry of ligand and receptor interaction is critical for constitutive activation of many signal transduction pathways involving enhances the expression level of its ligand, Jagged1, a finding that has not been previously reported in cancer cells. The presence of a positive regulatory loop helps explain why Jagged1 is co-upregulated with Notch3 in ovarian serous carcinoma tissues in which gene amplification is detected in the *Notch3* locus but not in the *Jagged1* locus. Furthermore, we also demonstrated that the Wnt/β-catenin pathway was essential to regulate Jagged1 expression, indicating that Jagged1 expression is regulated by at least two signaling pathways in ovarian carcinoma. The results from this study have several biological implications relevant to the study of the pathogenesis of ovarian cancer.

Using multiple approaches, including gene knockdown in ovarian cancer cells, ectopic expression in human OSE cells and induced Notch3 expression in mouse OSE and fallopian tube cells, we demonstrated that Notch3 signaling was essential and sufficient to upregulate Jagged1 expression. Although Notch signaling has been reported to increase levels of Jagged1 in NIH3T3 cells [10], the current report is the first to demonstrate that a similar phenomenon occurs in cancer cells. The biological significance of a Jagged1/Notch3 positive auto-regulatory loop in cancer cells is open to question. It is well-known that Notch signaling is involved in embryonic organ development and adult tissue differentiation/regeneration [11]. Studies from C. elegans and Drosophila suggest a role of Notch signaling in lateral inhibition or lateral specification. For example, during sensory organ development, precursor cells express both Notch receptor and its ligand Delta in the same cells; however, in adult tissue, Notch receptor and ligand are expressed by different tissue types [12]. In the tumorigenesis of human cancer, findings by us and others have demonstrated that a significant number of ovarian, prostate, and lung carcinomas co-express both Notch receptor and ligand [8, 13, 14]. This indicates either that tumor is derived from a small number of precursor cells which co-express both Notch receptor and ligand, or that during tumor development cancer cells may deregulate the tight control of gene expression to permit expression of both Notch receptor and ligand in the same cell. Nevertheless, a positive auto-regulatory loop supports co-expression of Notch and its ligand is likely to amplify or sustain Notch3 signaling activation, thus providing a long-term survival advantage for tumor cells. Because Jagged1 has been known to participate in vascular development [15], it would be interesting to determine if this positive regulatory loop in cancer cells is also involved in promoting vasculogenesis in the tumor microenvironment.

This report also demonstrated that the Wnt/β-catenin pathway may serve as a second pathway to ensure robust expression of Jagged1 in some ovarian serous carcinomas. Evidence that the Wnt signaling pathway regulates Jagged1 expression has been reported previously in progenitor cells and in colorectal cancer cells [16, 17]. Jagged1 was shown to be a direct target gene of β-catenin/TCF. The Jagged1 promoter contains two TCF consensus binding sites, and specific binding of β-catenin to this locus was demonstrated by ChIP. In findings similar to ours, suppressing the Wnt signaling pathway by dominant-negative TCF or small molecular inhibitor for β-catenin results in reduced Jagged1 expression. Our findings further suggest that regulation of Jagged1 expression by Wnt signaling is probably a common phenomena shared by most epithelial cancer cells.

Ovarian cancer has dual signaling pathways to control Jagged1 expression indicates the importance of maintaining active Notch signaling in epithelial cancer cells. In addition, the regulation of Jagged1 expression by both Wnt/β-catenin and Notch3 pathways may have a biological effect beyond being engaged in activating Notch3 pathway. Jagged1 may have signaling functions that are independent of the canonical Notch pathway. It has been demonstrated that upon binding to Notch receptor, Jagged1 is sequentially processed by α- and γ-secretase, leading to the release of the nuclear signaling fragment of Jagged1 [18, 19]. Similar to Notch, the soluble Jagged1 intracellular fragment translocates into the nucleus and activates gene expression via the transcription factor AP1 [18]. The putative Notch-independent role for Jagged1 in human neoplasms is not clear, and our results did not demonstrate that Jagged1 upregulation affected the expression level of Notch3, suggesting that the autoregulatory loop was “one way”—from Notch3 to Jagged1. Interestingly, we observed that the Jagged1 intracellular domain fragment increased much more than did total Jagged1 in OSE cells after transduction with NICD3. Further investigation into the function of the Jagged1 intracellular domain fragment will be necessary to delineate any putative Notch-independent function of Jagged1 in cancer cells.

In summary, this study provides evidence for a dual regulation of Jagged1 expression by Wnt and Notch signaling pathways in ovarian cancers. These results suggest that combinatorial therapy targeting both Notch and Wnt pathways may prove to be more effective than a single agent for treatment of epithelial cancers.

## MATERIALS AND METHODS

### Quantitative real-time PCR

Relative mRNA transcript expression levels were measured by quantitative real-time PCR using a previously described method. The primer sequences are listed in supplementary Table S1. Average fold changes were calculated by differences in threshold cycles (*C*_t_) between pairs of samples to be compared. The β-amyloid precursor gene (*APP*) was used for normalizing the cDNA concentration of each sample.

### Western blot analysis

Protein lysates were prepared by resuspending cell pellets in Laemmli sample buffer containing 5% β- mercaptoethanol. Protein lysates were separated by 4% to 12% or 4% to 20% Tris-glycine gel electrophoresis and transferred to polyvinylidene difluoride membranes using a semidry apparatus (Bio-Rad). Antibodies used in this study include anti-NICD3 (Santa Cruz Biotechnology), anti-Jagged1 (Santa Cruz Biotechnology). After incubation with horseradish peroxidase (HRP)-conjugated secondary antibody, signals were examined by enhanced chemiluminescence solution (Thermo Scientific).

### Generation of shRNA lentivirus

Notch3, RBPj-k, β-catenin, and TCF4 small hairpin RNA(shRNA) were purchased from Sigma-Aldrich. Notch3 shRNA4 template (CCGGCCAATGCCAACTGAAGAGGATCTCGAGATCCTCTTCAGTTGCATTGGTTTTT); Notch3 shRNA5 template (CCGGCCAGTTCACCTGTATCTGTATCTCGAGTACAGATACAGGTGAACTGGTTTTT); Jagged1 shRNA1 template (CCGGCCGAATGGAGTACATCGTATACTCGAGTATACGATGTACTCCATTCGGTTTTTG); Jagged1 shRNA2 template (CCGGCCAGGATAACTGTGCGAACATCTCGAGATGTTCGCACAGTTATCCTGGTTTTTG); RBPjk shRNA1 template (CCGGGCTGGAATACAAGTTGAACAACTCGAGTTGTTCAACTTGTATTCCAGCTTT TT); RBPjk shRNA3 template (CCGGGCACAGATAAGGCAGAGTATACTCGAGTATACTCTGCCTTATCTGTGCTTTTT); β-catenin shRNA3 template (CCGGCCTTTAGCTGTATTGTCTGAACTCGAGTTCAGACAATACAGCTAAAGGTTTTT); β-catenin shRNA5 template (CCGGCCATTGTTTGTGCAGCTGCTTCTCGAGAAGCAGCTGCACAAACAATGGTTTTT); TCF4 shRNA2 template (CCGGGCAGACATCAATTCCAGTCTTCTCGAGAAGACTGGAATTGATGTCTGCTTTTT); TCF4 shRNA3 template (CCGGCGAATTGAAGATCGTTTAGAACTCGAGTTCTAAACGATCTTCAATTCGTTTTT) were cloned into pLKO.1 vector respectively. The packaging plasmid psPAX2 and envelope plasmid pMD2.G were co-transfected with shRNA plasmid into 293FT cells for lentivirus generation. Supernatants from the 293FT culture were harvested for lentivirus two days after transfection and were used to transduce ovarian cancer cells.

### Production of NICD3 retroviruses

The NICD3 plasmid expressing the active intracellular fragment of Notch3 [20] and was cloned into pBabe retrovirus backbone with V5 tag. Packaging cells (Phoenix cells) were transiently transfected with NICD3 or empty vector using lipofectamine method (Invitrogen). Two days later, the viral supernatant was collected and polybrene was added to 8 μg/mL for retrovirus transduction.

### Cell growth and colony formation assays

Cells were grown in 96-well plates at a density of 5,000 per well. Cell number was measured by the incorporation of SYBR green I nucleic acid gel stain (Molecular Probes) using a fluorescence microplate reader (Fluostar from BMG). Data were determined from five replicates at different time points. For colony formation assay, cells were seeded into 25-cm2 flasks at a cell density of 2,000 cells per flask. After two weeks, colonies were stained with crystal violet (Sigma) and counted.

### Generation of Notch3 inducible mice

Human full-length Notch3 cDNA was inserted into the ROSA26 locus in embryonic stem cells by homologous recombination [21]. Notch3 was first cloned into pBIG-T and shuttled into pROSA26PA [21] prior to transfection into ES cells. The targeted locus includes a 5' floxed stop/polyA cassette fused to the expression cassette; the floxed cassette prevents expression in the absence of Cre recombinase. The Cre/Lox reaction activates expression of the Notch3 cassette. The primer sequences for genotyping were: forward 5'-CAGCTGTCACAGCCGATGCCC-3', reverse: 5'-AGCAGCTTGGCAGCCTCATAG C-3', which will amplify a 340 band for Notch3 knockin allele. The ROSA locus primers are forward 5'-CGTGATCTGCAACTCCAGTC-3', reverse 5'- GGAGCGGGAGAAATGGATATG-3', which will amplify a 425 bp band in wildtype allele, but will not produce a PCR product in knockin allele since this locus is disrupted. Control primers that amplify TCRD locus were: forward 5'- CAAATGTTGCTTGTC TGGTG-3', reverse 5'- GTCAGTCGAGTGCACAGTTT- 3'.

## SUPPLEMENTAL TABLES

Supplemental Table 1
