# Supplementary material for: Jagged1 Expression Regulated by Notch3 and Wnt/β-catenin Signaling Pathways in Ovarian Cancer
Source: Oncotarget. 2010 Jul 23;1(3):210–8. doi: 10.18632/oncotarget.127 (PMC2953807; doi:10.18632/oncotarget.127)
Supplement: Supplementary file 1 [file oncotarget-01-210-s001.docx]

Table S1 Xu et al

Supplementary Table 1. Sequences of PCR primers used in this study.

Gene Symbol	Forward primer	Reverse primer
NOTCH3	GTGTGTGTCAATGGCTGGAC	GTGACACAGGAGGCCAGTCT
JAG1	ATCGTGCTGCCTTTCAGTTT	GGTCACGCGGATCTGATACT
RBPJ	GGCAACAGTGATGACATTGG	GCTGTGAACTGGCATGAAAA
HES1	CGGACATTCTGGAAATGACA	TACTTCCCCAGCACACTTGG
PBX1	CGGAGCTGGAGAAATACGAG	CTGTCGCTTGCTTGTTGAAA
CTNNB1	TCGAGGACGGTCGGACT	ATTGTCCACGCTGGATTTTC
CCND1	TCCTCTCCAAAATGCCAGAG	GGCGGATTGGAAATGAACTT
SOX9	GACGCTGGGCAAGCTCT	GTAATCCGGGTGGTCCTTCT
APP	GTGAAGATGGATGCAGAATTCCG	AAAGAACTTGTAGGTTGGATTTTCG
